# Effects of yeast beta-1,3/1,6-glucans on nutrient digestibility, intestinal functionality, and immune and antioxidant variables in growing dogs submitted to spay or neutering surgery

**DOI:** 10.1371/journal.pone.0331843

**Published:** 2025-09-08

**Authors:** Renata Bacila Morais dos Santos de Souza, Eduarda Lorena Fernandes, Lorenna Nicole Araújo Santos, Laiane da Silva Lima, Heloísa Lara Silva, Thaila Cristina Putarov, Simone Gisele de Oliveira, Ananda Portella Félix

**Affiliations:** 1 Department of Animal Science, Federal University of Paraná, Curitiba, Paraná, Brazil; 2 Biorigin (Açucareira Quatá S.A.), Lençóis Paulistas, São Paulo, Brazil; South China Agricultural University, CHINA

## Abstract

This study aimed to assess the impact of yeast beta-1,3/1,6-glucans (BG) on apparent digestibility coefficients (ADC) of nutrients, intestinal fermentative metabolites, fecal microbiota profile, and immune and antioxidant variables in puppies before and after surgical challenge. Two treatments were evaluated: control, without, and test, with oral supplementation of 65 mg/kg body weight/day of purified BG from *Saccharomyces cerevisiae* for 120 days. For this, 16 growing Beagle dogs were distributed in a completely randomized design (n = 8/treatment). On day 31, dogs were submitted to spay or neutering surgery. Diet ADC and fecal characteristics analyses were performed on days 55–60. Fecal (days 0, 15, 30, 34, and 60) and blood (days 0, 30, 34, and 60) samples were collected to evaluate intestinal fermentative metabolites, fecal IgA and microbiota, intestinal permeability, and immune and antioxidant variables. On day 80, all dogs were vaccinated for rabies and blood samples were collected on day 120 to determine antibody titers. The supplementation of BG promoted an increase in fecal IgA concentrations on day 15 (P < 0.05) and an increase in fecal concentrations of butyrate (P < 0.05) when day 30 minus day 0 were compared. Dogs of the BG group presented higher fecal concentrations of serotonin (day 15), spermidine (days 15, 30, and 34), and a reduction in tyramine, histamine, and cadaverine on day 60 (P < 0.001). BG consumption promoted an increase in richness and a clear differentiation in the fecal microbiota profile on days 34 and 60 (P < 0.05). BG group also presented an increase in fecal *Faecalibacterium*, *Blautia*, and *Turicibacter* on day 34 (P < 0.05). Reduced glutathione and catalase activities were higher in the BG group (P < 0.05), regardless of the day. In conclusion, the supplementation of BG does not alter the ADC of nutrients, beneficially modulates the intestinal functionality, and stimulates the activity of antioxidant enzymes in growing dogs submitted to a surgical challenge.

## Introduction

During the growth phase, dogs are challenged with a series of stressors, including separation from the dam, dietary changes, and veterinary procedures, including, in some cases, surgeries such as castration. These challenges can disrupt the immune system, leading to an imbalance in antioxidant and anti-inflammatory mechanisms [1] and alterations in the gut microbiota profile [2]. This is particularly concerning at this phase since their immune system and gut microbiota are still developing [3].

In this context, well-balanced diets associated with some feed additives are important to commensal gut microbiota establishment and maturation of the immune system in the growing phase. Among these additives are beta-glucans, polysaccharides that are the main structural components of the cell wall of yeasts, fungi, some bacteria, and cereals [4]. Specifically, purified beta-1,3/1,6-glucans derived from the yeast *Saccharomyces cerevisiae* (BG) have been studied and recognized for their immunomodulatory activity in dogs [5,6]. This BG can activate the immune system by binding to macrophage receptors and modulating the inflammatory response [7], possibly modulating the intestinal microbiota due to their relationship with mucosa-associated lymphoid tissue.

In the literature, the effects of BG have been demonstrated in humans, rats, and other animal species [4,8,9]. In adult dogs, despite the limited publications evaluating the purified form of BG, some evidence has demonstrated its potential benefits. This includes a reduction in inflammatory response in dogs with chronic inflammatory enteropathy [5], as well as modulation of innate and adaptive immunity and an increase in bacterial genera associated with eubiosis in healthy dogs supplemented with BG [10,11]. However, no studies have evaluated the effects of oral BG supplementation on these variables in growing dogs undergoing a surgical challenge.

Therefore, we hypothesize that oral administration of BG could be beneficial for puppies undergoing post-surgical stress, possibly modulating the immune system and minimizing the alterations in the gut microbiota caused by surgery. In this context, the objective was to evaluate the effects of purified BG on digestibility of nutrients and energy, fecal characteristics, intestinal fermentative metabolites and permeability, fecal microbiota, and immune and antioxidant markers in puppies undergoing spay or neutering surgery.

## Materials and methods

The Ethics Committee on Animal Use of the Agrarian Sciences Sector of the Federal University of Paraná, Curitiba, PR, Brazil, approved the use of animals for this study under protocol n. 037/2022. The study was conducted at the Research Laboratory on Canine Nutrition-LENUCAN in Curitiba, Paraná, Brazil (25º 25’ 40” S, 49º 16’ 23” W).

### Animals and facilities

Sixteen growing Beagle dogs (8 males and 8 females) with an initial age of 3 months (106.25 ± 17.9 days) and a mean body weight of 4.06 ± 0.50 kg were used. The animals were submitted to clinical examination before and after the experimental period.

The dogs were individually housed in brickwork kennels (5 m long x 2 m wide), containing a bed and free access to fresh water. During most of the experimental period, the dogs had free access to an outdoor area of 1,138 m^2^ for 4 h/day for voluntary exercise and socialization. During the feces collection period, the dogs were individually housed in kennels. The facilities had side wall bars that allowed visual and limited interaction with neighboring dogs. Besides, the animals received extra attention and environmental enrichment inside the kennel during this period. The temperature ranged from 16°C to 28°C, with a 12h light-dark cycle (light from 6:00 am to 6:00 pm). The dogs were supervised by the researchers and the veterinarian responsible for the laboratory throughout the experimental period. After the experimental period, all dogs remained housed in the institutional experimental kennel facility, where they continue to be maintained and monitored according to institutional care protocols.

### Experimental groups

Two treatments were evaluated: Control, without the feed additive, and BG, with oral supplementation of 65 mg/ kg body weight/ day (as-fed basis) of beta-1,3/1,6-glucans purified from the yeast *Saccharomyces cerevisiae* (MacroGard®, Açucareira Quatá S.A-Biorigin, Lençóis Paulista, SP, Brazil). The product contained 78% BG, according to the manufacturer and was weighed daily and individually on a precision scale (Analytical electronic scale, model FA2104N, 0.0001 g, Bioprecisa®, PR, Brazil), and offered to the dogs on top of the diet at the time of feeding, ensuring the right amount for each animal.

The dogs consumed the treatments for 120 days. The animals were weighed weekly and fed twice a day (8:00 a.m. and 4:00 p.m.) according to their metabolizable energy requirements (MER) for growth recommended by the European Pet Food Industry Federation [12] and adjusted according to the growth standard charts proposed for healthy puppies [13]. The equation used was: MER (kcal/day) = 210−135 (adjusted according to the growth standard chart) × [actual body weight (kg)/expected mature body weight (kg)] × actual weight (kg)^0.75^ [12], considering that the weight of an adult Beagle is 11 kg.

The basal diet for the experiment (used for both the control and BG groups) was an extruded complete dry food for puppies. The diet contained the following ingredients: meat and bone meal, broken rice, poultry by-product meal, corn, wheat bran, corn gluten meal, poultry fat, hydrolyzed chicken and pork liver, sodium chloride, sugar, propionic acid, BHT (butylated hydroxytoluene), and vitamins and minerals premix. The diet did not contain yeast or its derivatives, or other functional additives that could interfere with the animal’s intestinal functionality such as prebiotics or probiotics. Water was provided *ad libitum*. The chemical composition of the diet is described in Table 1.

**Table 1:** Analyzed chemical composition (%, dry matter basis) of the basal diet.

Item	%, Dry matter basis
**Dry matter**	94.02
**Crude protein**	24.16
**Acid-hydrolyzed ether extract**	11.74
**Total dietary fiber**	6.43
**Ash**	10.18
**Calcium**	2.09
**Phosphorus**	1.62
**Gross energy (kcal/kg)**	4637.20

### Nutrient digestibility, metabolizable energy and fecal characteristics

The digestibility trial followed the total fecal collection method recommended by the Association of American Feed Control Officials [14], with five days of total fecal collection (days 55–60 of the experiment). The food was offered during a 55-day adaptation period followed by 5 days of total fecal collection. The animals were fed twice a day (08:00 a.m. and 04:00 p.m.) in amounts sufficient to supply the MER of growing dogs as recommended by the European Pet Food Industry Federation [12], as described before. Feces were collected at least twice a day, weighed, stored in individual plastic bags previously identified, covered, and stored in a freezer at −14°C to be analyzed later. At the end of the collection period, the feces were thawed at room temperature and homogenized separately, forming a composite sample of each animal.

Feces were dried in a forced ventilation oven (320-SE, Fanem, São Paulo, Brazil) at 55°C for 72h or until reaching a constant weight. After drying, the feces and the experimental diet were ground using a 1 mm sieve in a grinder (Arthur H. Thomas Co., Philadelphia, PA, USA) and analyzed for dry matter (DM) at 105°C for 12h; nitrogen (N, method 954.01), and crude protein (CP), which was calculated as N × 6.25; ash (method 942.05); and acid-hydrolyzed ether extract (AEE) (method 942.05). All analyses followed the recommendations of the Association of Official Analytical Chemists [15]. The total dietary fiber of the diet was analyzed according to [16]. Gross energy (GE) was determined in a calorimeter pump (IKA C2000 Basic, IKA-Werke, Staufen, Germany).

Fecal characteristics were evaluated during the collection period by total dry matter (DMf) content and consistency by score. An aliquot of feces (2 g) was dried at 105°C for 48 hours to determine DMf. Fecal score was always evaluated by the same researcher, who assigned points from 1 to 5, being: 1 = feces are soft and have no defined shape; 2 = feces are soft and poorly formed; 3 = feces are soft, formed, and moist; 4 = feces are well-formed and consistent; and 5 = feces are well-formed, hard and dry [17].

Fecal pH was analyzed in feces collected up to 15 min after spontaneous defecation on days 0, 15, 30, 34, and 60 of the experiment, and was measured using a digital pH meter (331, Politeste Instrumentos de Teste Ltda., São Paulo, SP, Brazil). For this, 3 g of fresh feces were diluted in 30 mL of distilled water.

### Fecal IgA, intestinal fermentative metabolites, and fecal microbiota

Fresh stool samples (up to 15 min after defecation) were collected for IgA, fermentative metabolites, and microbiota analysis on days 0, 15, 30, 34, and 60 of the experiment. Fecal concentrations of IgA were analyzed according to [18]. Fecal ammonia concentrations were determined according to [19]. Briefly, 5 g of fresh feces were incubated in a 500 mL lidded glass balloon, containing 250 mL distilled water, for 1 h. Then, three drops of octyl alcohol (1-octanol) and 2 g of magnesium oxide were added to the solution, subsequently distilled in a Macro-Kjeldahl apparatus, and recovered in a beaker containing 50 mL boric acid. Finally, ammonia was titrated using standardized sulphuric acid at 0.1 N.

For determination of short-chain (SCFA, acetate, butyrate, propionate, and valerate) and branched-chain (BCFA, isovalerate and isobutyrate) fatty acids, fresh feces were collected up to 15 min after defecation. In a plastic container with a lid, 10 g of stool sample was weighed and mixed with 30 mL of 16% formic acid. This mixture was homogenized and stored in a refrigerator at 4°C for a period of 3–5 days. After this period, the solutions were centrifuged at 2500 gx (2K15, Sigma, Osterode am Hans, NI, Germany) for 15 min. At the end of centrifugation, the supernatant was separated and subjected to further centrifugation. Each sample underwent three centrifugations, and at the end of the last one, part of the supernatant was transferred to a properly labeled Eppendorf tube for subsequent freezing at –14°C. Afterward, the samples were thawed and underwent new centrifugation at 18000 gx for 15 min (Rotanta 460 Robotic, Hettich, Tuttlingen, BW, German). Both centrifugations were conducted under refrigeration (approximately 5°C). Fecal SCFA and BCFA were analyzed by gas chromatography (Shimadzu, model GC-2014, Kyoto, Honshu, Japan), using a glass column (Agilent Technologies, HP INNO wax – 19,091 N, Santa Clara, CA, United States of America) 30 m long and 0.32 mm wide. The injected volume of the supernatant was set to 1 μL. Nitrogen was used as the carrier gas with a flow rate of 3.18 mL/min. The working temperatures were 200°C at the injector, 240°C at the column (at a speed of 20°C/min), and 250°C at the flame ionization detector.

The mean percentage of phenols, indoles, and p-cresol concentration peaks in the feces were analyzed by chromatography, with a GCMS2010 Plus gas chromatographer (Shimadzu, Kyoto, Japan®), coupled to a TQ8040 mass spectrometer with an AC 5000 autosampler and a split–splitless injector. Chromatographic separations were obtained in the SH-Rtx-5MS (30m × 0.25 mm × 0.25 µm—Shimadzu®) column with a 1mL/min flow rate, and helium 5.0 (White Martins) as the carrier gas. The transfer line and ionization source temperatures were maintained at 40°C and 220°C, respectively, with an injection volume of 1L in split mode (ratio 1:10). The GC oven temperature was maintained at 220°C for 5 minutes, with a 40°C^-1^ increase to 280°C (5 min). The total analysis time was 31 min, and the mass spectrometer operated in full scan mode (m/z = 40–400) and selective ion monitoring (SIM) modes, with electron ionization at 70 eV. The software used in the data analysis was the GCMS solution® (version 4.30, Shimadzu, Kyoto, Japan). Biogenic amines were analyzed according to the method described by [20].

For evaluation of the fecal microbiota, approximately 2 g of sample was taken from the interior of the freshly collected stool, placed in a sterile Eppendorf tube, and stored in a – 80°C freezer until the moment of the analysis. For DNA extraction from the samples, the commercial kit “ZR Fecal DNA MiniPrep®” from Zymo Research (Zymo Research, Irvine, CA, USA) was used following the protocol recommended by the manufacturer.

The DNA extracted was quantified by spectrophotometry at 260 nm using the NanoDrop® 2000 spectrophotometer (Thermo Scientific, Wilmington, VA, USA). To evaluate the integrity of the extracted DNA, all samples were run by electrophoresis in a 1% agarose gel, stained with a 1% ethidium bromide solution, and visualized with ultraviolet light in a transilluminator. A 460-base segment of the V4 hypervariable region of the 16S rRNA gene was amplified using the universal primers 515F and 806R and the following polymerase chain reaction (PCR) conditions: 94°C for 3 min; 18 cycles of 94°C for 45s, 50°C for 30s, and 68°C for 60s; followed by 72°C for 10 min. From these amplifications, a metagenomic library was built using the commercial Nextera DNA Library Preparation Kit from Illumina® (San Diego, CA, USA). The amplifications were pooled and afterward sequenced in the Illumina® “MiSeq” sequencer [21]. The obtained reads were analyzed on the Quantitative Insights into Microbial Ecology (QIIME) platform [22,23], followed by a workflow of low-quality sequences removal, filtration, chimera removal, and taxonomic classification. The sequences were classified into bacterial genera by recognizing amplicon sequence variants (ASVs), in this case, the homology of the sequences when compared against a database. To compare the sequences, the GTDB 202 update for 2021 of the Genome Taxonomy Database ribosomal sequence database was used [24]. In order to normalize the data and not compare samples with different numbers of reads, 12,293 reads per sample were used to generate the classification of bacterial communities by ASV identification.

### Blood analysis

Blood was collected on days 0, 30, 34, and 60 of the experiment. Before the collections, the dogs were submitted to a 12-hour fasting period. After physical contention and antisepsis with 70% alcohol on the ventral region of the neck, 5 mL of blood was collected by jugular venipuncture. Blood samples for oxidative stress analysis were collected using heparinized syringes and placed in a tube with citrate. The samples for inflammatory variables analysis and intestinal permeability were collected using non-heparinized syringes and placed in a tube without anticoagulant.

#### NF-κB and antioxidant enzymes.

Quantitative detection of canine NF-κB p65 in serum was measured using a specific ELISA kit (Cat No. MBS2608289) according to the manufacturer’s protocol (MyBioSource, Inc, San Diego, CA, USA). Serum antioxidant markers were evaluated by measuring superoxide dismutase (SOD), catalase (CAT), reduced glutathione (GSH), lipid peroxidation (LPO), and plasmatic proteins.

For SOD and CAT analysis, samples were homogenized in a potassium phosphate buffer solution (pH 6.5) at 1:10 dilution and centrifuged at the speed of 10000 g for 20 min under 4°C. SOD activity was quantified through its ability to inhibit the autoxidation of the pyrogallol reagent [25]. CAT activity was quantified according to [26].

GSH levels were measured by the [27] technique. Samples were homogenized in a potassium phosphate buffer (pH 6.5) at 1:10 dilution. Subsequently, 100 µL was mixed with trichloroacetic acid (80 µL, 12.5% purity grade). The supernatant was separated by centrifugation at 3000 g for 15 min at 4°C. The LPO rate was measured by the ferrous oxidation-xylenol orange (FOX) method [28]. This method quantifies the formation of hydroperoxides during lipid peroxidation, as hydroperoxides oxidize iron to ferric ions, which, in turn, bind to the xylenol orange dye. Quantification of plasmatic proteins in samples was done in microplates [29] using bovine albumin as a standard. The sample (10 µL homogenized in a 6.5 pH potassium phosphate buffer, centrifuged at 10000 g under 4°C for 20 min, and diluted at a 1:10 proportion) was used in each well of the microplate. Then, it was reacted with 250 µL of Bradford’s solution. The reading was performed in a microplate reader at 595 nm. The value measured for protein, expressed in mg, was used to calculate the previous parameters.

#### Intestinal permeability.

Intestinal permeability was assessed using fluorescein dextran isothiocyanate marker (FITC-d, 3–5 kDa). The marker was administered orally (1 mL/dog) to fasting dogs. After 6 hours, blood was collected and analyzed by fluorescence in the serum [30].

### Spay and neutering surgery

Dogs were submitted to castration surgery under total anesthesia on day 31 of the experiment. This surgery was planned as a challenge to the organism, considering that the animals would already be castrated at the same age.

Surgical procedures were performed at the Department of Veterinary Medicine in the Veterinary Hospital of the Federal University of Paraná (Curitiba, Brazil), after a 12-hour fasting period. Standardized ovariohysterectomy (OVH) procedure for females, and Orchiectomy (OHE) for males was performed. The anesthesia protocol was similar for all dogs and performed by the same surgeon and anesthesiologist. There were 4 days of surgery, each day four dogs (two from the control and two from the BG group) underwent the procedure. Day 34 of the experiment was different for each group of animals and was determined by the day of surgery.

The animals were premedicated with acepromazine (0.02 ml/kg body weight) (Acepran® 0.2%, Vetnil, São Paulo, Brazil) and methadone (0.3 mg/kg body weight) (Mytedom® 1%, Cristália, São Paulo, Brazil). Anesthesia was induced with propofol (3–5 mg/kg body weight) (Propotil® 1%, BioChimico, Rio de Janeiro, Brazil). The surgical technique of OVH was performed according to [31] and the OHE technique was the conventional open castration by the standard pre-scrotal method [32].

During surgery, the dogs were maintained in inhalational anesthesia with isoflurane (Isoforine®, Cristália, São Paulo, Brazil) at 1.3 MAC (Minimum Alveolar Concentration), through a universal vaporizer, dosed by bubbling. Lidocaine (0.2 ml/kg body weight for males and 0.8 ml/kg for females) (Lidovet® 2%; Bravet, Rio de Janeiro, Brazil) was used. Ringer’s lactate solution (Ringer with lactate; JP Farma; Ribeirão Preto, Brazil) was infused at a rate of 5 ml/kg/hr until the end of anaesthesia.

Postoperatively, the animals received an injectable single dose of meloxicam (0.1 mg/kg body weight) (Elo-xicam® 0.2%, Chemitec, São Paulo, Brazil), an injectable single dose of dipyrone (25 mg/kg body weight) (Febrax®, Lema-Injex, Vespasiano, Brazil) and sodium cephalothin (30 mg/kg body weight) (Cefariston®, Blau, São Paulo, Brazil).

Post-operative drugs for all dogs were amoxicillin with potassium clavulanate (Agemoxi 250 mg®; Agener União; São Paulo, Brazil) – 25 mg/kg orally, once daily, dipyrone (Dipirona sódica 500 mg/ml; EMS; Hortolândia, Brazil) – 25 mg/kg orally every 8 h for 5 days, and meloxicam (Meloxinew® 0.5 mg; Vetnil; São Paulo, Brazil) – 0.1 mg/kg orally, once daily, for 3 days.

### Serum neutralizing antibody titers

To determine the influence of oral administration of BG on the immune response, antibody titration was carried out after vaccination. On day 80 of the experiment, the animals received one subcutaneous injection with the inactivated rabies vaccine (NOBIVAC® Rabies; MSD Saúde Animal; São Paulo; Brazil). After 5 weeks, blood samples were collected from all the dogs via jugular venipuncture.

Titration of rabies virus neutralizing antibodies (VNAs) was performed by using the fluorescent antibody virus neutralization (FAVN) test according to the technique described by [33] with a positive threshold of 0.5 IU/ml.

### Calculations and statistical analysis

The organic matter (OM) was calculated by: 100 – ash. The apparent digestibility coefficients (ADC) and ME were estimated according to [14], based on the equations: ADC = (g nutrient intake – g nutrient excreted)/g nutrient intake; and ME (kcal/kg) = {kcal/g GE intake – kcal/g fecal GE – [(g CP intake – g fecal CP) × (1.25 kcal/g)]}/g feed intake.

Data were analyzed for normality by the Shapiro-Wilk test. Data with time effect (fecal pH, intestinal fermentative metabolites, and blood parameters) and normal distribution were submitted to analysis of covariance (ANCOVA) in a split-plot design (n = 8), using day 0 as the co-variable. Only the blood variables presented significant co-variable (P < 0.05). When an effect of day or interaction (treatment × day) was observed, means were compared by Tukey’s test. The differences (final – initial) among the experimental days and digestibility data were analyzed by the Student t-test (P < 0.05). Fecal score data were analyzed by Wilcoxon test (P < 0.05), and biogenic amines were analyzed by Kruskal-Wallis test (P < 0.05). P-values greater than 0.05 and lower than 0.10 were considered a tendency.

Data of alpha-diversity indexes (Chao1 and Shannon) of the microbiota were analyzed using the Kruskal-Wallis test (P < 0.05). Linear discriminant analysis (LDA) of effect size (LEfSe) was applied to verify the bacterial genera with the greatest discriminatory power between treatments on each day. Adjusted P-values for *false discovery rate* <0.05 and LDA scores greater than 2 were considered. Beta-diversity was analyzed by Principal Coordinate Analysis (PCoA), using the Bray-Curtis dissimilarity method. Differences between groups were analyzed using the PERMANOVA test, considering P < 0.05. All the analyses were conducted using Minitab 18 statistical software program (Minitab® Inc., USA) and MicrobiomeAnalyst 2.0 [34].

## Results

### Nutrient digestibility, metabolizable energy and fecal characteristics

No adverse effects on diets were observed. The BG supplementation did not alter the diet ADC of nutrients and energy and ME (P > 0.05; Table 2). The DMf, feed intake, and fecal production and score did not differ between treatments (P > 0.05; Table 2).

**Table 2:** Means of feed intake (dry matter basis), apparent digestibility coefficients (ADC), metabolizable energy (ME), and fecal characteristics of dogs fed without (control) or with BG supplementation.

	Treatments^*^		SEM^¹^	P^2^
Item	Control	BG		
**Feed intake (g/dog/day)**	317.83	333.4	12.79	0.279
**ADC (%)**				
**Dry matter**	71.8	71.8	0.85	0.495
**Organic matter**	75.7	75.7	0.74	0.492
**Crude protein**	75.1	76.3	1.00	0.288
**Acid-hydrolyzed ether extract**	84.1	83.0	0.72	0.280
**Gross energy**	76.8	76.5	0.74	0,392
**ME (kcal/kg)**	3540.7	3523.4	34.34	0.388
**Fecal characteristics**				
**Dry matter (%)**	28.89	28.19	0.568	0.308
**Prodution³ (g/day)**	304.03	326.97	9.562	0.114
**Score**	3.3 (2.8/4.0)	3.0 (3/3.5)	–	0.409

^1^SEM = standard error of mean; ^2^P = Probability by Student’s t-test (P < 0.05); ^3^Production = g feces produced as-is/animal/day. ^4^Score: Median (1˚/3˚quartiles) analyzed by Wilcoxon test (P* < *0.05). ^*^n = 8/treatment.

The BG group presented a tendency to lower fecal pH when compared to the control group (P = 0.053), but there was no effect of days on this variable (P > 0.05, Table 3). Fecal ammonia increased on days 15, 34, and 60, when compared to day 0, regardless of treatments (P < 0.05, Table 3).

**Table 3:** Means of fecal concentrations of ammonia and pH of dogs fed without (control) or with BG supplementation on days 0, 15, 30 (before surgery), 34 (after surgery), and 60.

Treatments^*^	Day	pH	Ammonia (μmol/g)
**Main factors**			
**Control**		6.76	0.19
**BG**		6.54	0.19
	0	6.63	0.15b
	15	6.67	0.22a
	30	6.49	0.17ab
	34	6.88	0.20a
	60	6.56	0.21a
**Interaction**			
**Control**	0	6.64	0.15
	15	6.80	0.22
	30	6.86	0.15
	34	6.89	0.21
	60	6.59	0.21
**BG**	0	6.62	0.14
	15	6.54	0.22
	30	6.12	0.19
	34	6.88	0.19
	60	6.54	0.21
**SEM** ^1^		0.057	0.007
**P** ^**2**^ **-Treatment**		0.053	0.833
**P** ^**2**^ **-Day**		0.213	0.001
**P** ^**2**^ **-Treatment x Day**		0.180	0.643

^1^SEM = standard error of mean; ^2^P = Probability; ^abc^Means with different letters differ according to Tukey’s test (P < 0.05); ^*^n = 8/treatment.

### Fecal IgA, intestinal fermentative metabolites, and fecal microbiota

**Fig 1:**
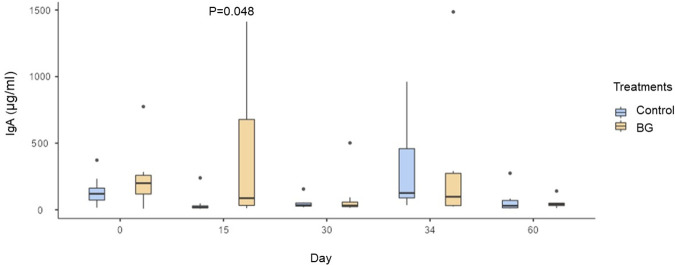
Mean values of fecal IgA concentrations (µg/ml) in dogs fed without (control) or with BG supplementation on days 0, 15, 30 (before surgery), 34 (after surgery), and 60. Fig 1. P = 0.048 on day 15 by Mann-Whitney test (P < 0.05).

There was an increase in fecal IgA concentrations in the BG group on day 15 compared to the control group (P < 0.05; Fig 1). However, there were no differences in this variable between groups on the other days (P > 0.05).

**Fig 2:**
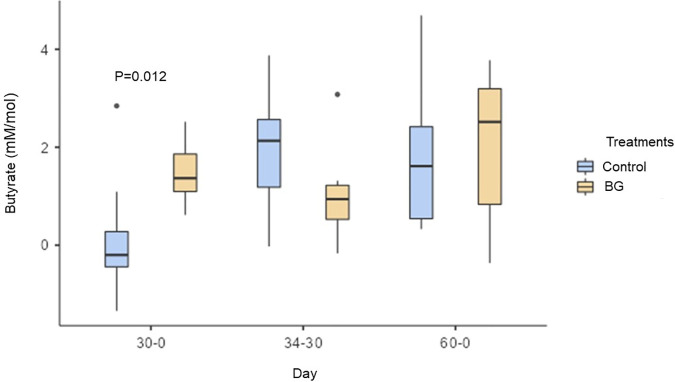
Variation (final – initial) of fecal concentrations of butyrate (mM/mol) in dogs fed without (control) and with BG supplementation on days 0, 15, 30 (before surgery), 34 (after surgery), and 60. Fig 2. P = 0.012 for day 30 − 0 variation by Student’s t-test between the control and BG groups.

Concerning SCFA, there was an increase in fecal concentrations of acetate on day 60 compared to days 15, 30, and 34, regardless of treatments (P < 0.05; Table 4). However, when evaluating the variation in fecal concentrations of acetate between day 30 and day 0, there was a tendency (P = 0.054) to higher acetate in feces of dogs from the BG group (day 30–0: control = −13.23 and BG = 3.18 mM/mol). On the other hand, fecal concentrations of propionate were higher on day 60 than on day 34 in all dogs, regardless of treatments (P < 0.05; Table 4). There was also no effect on the difference (final – initial) in fecal propionate concentrations between days (P > 0.05, not shown). In addition, there was an increase in fecal concentrations of butyrate on days 34 and 60 compared to day 0, regardless of treatment (P < 0.05; Table 4). When all days were considered, there was no difference in fecal concentrations of butyrate between the control and BG groups (P > 0.05; Table 4). However, when comparing the variation from day 30 minus day 0, only the BG group presented an increase in fecal concentrations of butyrate (P < 0.05; Fig 2).

**Table 4:** Means of fecal concentrations of short-chain (SCFA) and branched-chain (BCFA) fatty acids (mM/mol dry matter) of dogs fed without (control) or with BG supplementation on days 0, 15, 30 (before surgery), 34 (after surgery), and 60.

Treatments^*^	Day	Acetate	Propionate	Butyrate	Valerate	Total SCFA	Isovalerate	Isobutyrate	Total BCFA
**Main effects**									
**Control**		60.88	23.85	8.65	5.29	93.71	5.65	5.55	17.69
**BG**		59.27	23.72	8.36	5.29	91.24	5.6	5.57	17.67
	0	60.83ab	25.92ab	7.29c	5.29	94.04ab	5.59	5.58ab	17.68ab
	15	56.17b	22.86ab	8.40b	5.28	87.43b	5.66	5.57ab	17.74ab
	30	55.81b	22.58ab	8.10b	5.28	86.48b	5.52	5.43b	17.42b
	34	54.11b	19.54b	9.56a	5.31	83.72b	5.64	5.51b	17.66ab
	60	73.45a	28.015a	9.19a	5.31	110.72a	5.71	5.71a	17.92a
**Interaction**									
**Control**	0	64.98	27.25	7.62	5.3	99.86	5.72	5.6	17.84
	15	58.89	23.53	8.78	5.28	91.2	5.64	5.6	17.74
	30	51.75	20.71	7.77	5.27	80.24	5.52	5.4	17.38
	34	55.31	19.74	9.68	5.32	86.02	5.68	5.47	17.65
	60	73.44	28	9.38	5.31	111.24	5.7	5.67	17.86
**BG**	0	56.68	24.59	6.95	5.29	88.23	5.46	5.57	17.52
	15	53.45	22.18	8.02	5.28	83.66	5.69	5.55	17.74
	30	59.86	24.44	8.42	5.29	92.72	5.51	5.46	17.46
	34	52.92	19.33	9.45	5.29	81.41	5.61	5.55	17.67
	60	73.46	28.03	8.97	5.31	110.19	5.73	5.75	17.97
**SEM** ^1^		1.711	0.84	0.153	0.004	2.55	0.026	0.022	0.048
**P** ^2^ **-Treatment**		0.658	0.915	0.264	0.874	0.600	0.499	0.637	0.601
**P** ^2^ **-Day**		0.001	0.021	< 0.001	0.052	< 0.001	0.118	< 0.001	0.005
**P** ^2^ **-Treatment x Day**		0.518	0.791	0.429	0.324	0.570	0.276	0.702	0.570

^1^SEM = standard error of mean; ^2^P* *= Probability; ^abc^Means with different letters differ according to Tukey’s test (P < 0.05); ^*^n = 8/treatment.

Higher fecal concentrations of total SCFA were observed on day 60 compared to the other days, regardless of treatments (P < 0.05; Table 4). There was also a tendency (P = 0.056) for higher fecal concentrations of total SCFA in the BG group between days 30 and 0 (day 30–0: Control = −19.62 and BG = 4.50 mM/mol).

In general, there was an increase in fecal concentrations of isobutyrate and total BCFA on day 60 regardless of treatments (P < 0.05, Table 4). There was no variation effect (days 30−0, 34−30 or 60−0) on fecal concentrations of valerate and BCFA (P > 0.05, not shown).

Regarding biogenic amines, the BG group presented higher fecal concentrations of serotonin on day 15 compared to the control group on day 0 (P < 0.001, Table 5). Similarly, there was an increase in fecal concentrations of spermidine in the BG group on days 15, 30, and 34, compared to day 0 (P < 0.001, Table 5). The BG group also presented lower fecal concentrations of tyramine, putrescine, and histamine on day 60, compared to the control group on days 15–34 (P < 0.001, Table 5). There was also a significant reduction in fecal concentrations of cadaverine on day 60 compared to day 15 in BG group (P < 0.001, Table 5).

**Table 5:** Medians and interquartile range (IQR) of biogenic amines^1^ (mg/kg dry matter) in feces of dogs fed without (C) or with BG supplementation on days 0, 15, 30 (before surgery), 34 (after surgery), and 60.

Item^*^	0		15		30		34		60	
	C	BG	C	BG	C	BG	C	BG	C	BG
**Ser** ^¹^	19.3b	20.2b	25.2ab	47.0a	16.0b	21.9ab	26.8ab	22.4ab	19.9b	17.7b
**IQR**	4.65	6.04	10.3	9.72	3.17	5.36	5.78	8.92	4.56	2.43
**Tyr**	22.7abc	51.3ab	40.4ab	73.3ab	10.4bc	23.3abc	128.0a	134.0a	16.1abc	3.2c
**IQR**	15.9	19.1	79.9	34.1	20.8	40.2	120	135	38.5	1.04
**Spe**	52.2c	105.0abc	94.7abc	235.0ab	175.0abc	322.0ab	255.0ab	514.0a	85.3bc	126.0abc
**IQR**	18.2	51.3	69.5	137	164	177	176	447	88.5	30.9
**Cad**	105.0b	282.0ab	156.0ab	379.0a	131.0b	157.0ab	252.0ab	221.0ab	168.0ab	117.0b
**IQR**	112	147	269	108	6.94	131	97.4	132	45.9	19.2
**His**	6.3abc	12.3abc	14.6ab	25.3a	7.6abc	9.6ab	11.4ab	7.2abc	4.6bc	3.8c
**IQR**	7.19	5.81	13.4	24.9	5.29	16.5	10.4	6.07	1.76	0.62
**Put**	8.8bc	15.7abc	36.0abc	64.0ab	91.1a	45.6ab	91.2a	161.0a	9.2bc	7.1c
**IQR**	0.75	7.39	39.1	85.1	68.5	93.1	86.2	154	6.32	3.48

^1^Ser = serotonin; Tir = tyramine; Spe = spermidine; Cad = cadaverine; His = histamine; Put = putrescine; P < 0.01 for all variables by Kruskal-Wallis test; ^abc^Medians with different letters differ according to Dunn’s test (P < 0.05); ^*^n = 8/treatment.

There was no difference between the control and BG groups in the percentage of phenol, indole, and p-cresol peaks in feces (P > 0.05, Table 6). However, it was observed that, regardless of treatments, phenol peaks were higher on day 34 (after surgery) compared to the other days, and indole and p-cresol peaks were higher on days 15, 30, and 60 compared to day 0 (P < 0.001, Table 6).

**Table 6:** Means of peak areas (%) of indoles, phenol, and p-cresol of dogs fed without (control) or with BG supplementation on days 0, 15, 30 (before surgery), 34 (after surgery), and 60.

Treatments^*^	Day	Phenol	Indoles	P-cresol
**Main effects**				
**Control**		5.75	8.4	4.47
**BG**		4.81	8.94	4.15
	0	0.80b	1.68b	1.48c
	15	5.87b	12.70a	5.52ab
	30	5.23b	10.66a	7.31a
	34	11.55a	5.93ab	3.02bc
	60	2.94b	12.37a	4.19b
**Interaction**				
**Control**	0	0.97	2.54	1.65
	15	6.11	9.4	5.63
	30	4.77	12.99	7.93
	34	13.38	5.51	3.79
	60	3.17	11.57	3.33
**BG**	0	0.63	0.82	1.32
	15	5.63	16.01	5.41
	30	5.69	8.32	6.69
	34	9.36	6.36	2.24
	60	2.71	13.16	5.06
**SEM** ^¹^		0.696	0.922	0.354
**P** ^2^ **-Treatment**		0.468	0.804	0.373
**P** ^2^ **-Day**		<0.001	<0.001	<0.001
**P** ^2^ **-Treatment x Day**		0.667	0.243	0.439

¹ SEM = standard error of mean; ^²^Probability. ^abc^Means with different letters differ according to Tukey’s test (P < 0.05); ^*^n = 8/treatment.

**Fig 3:**
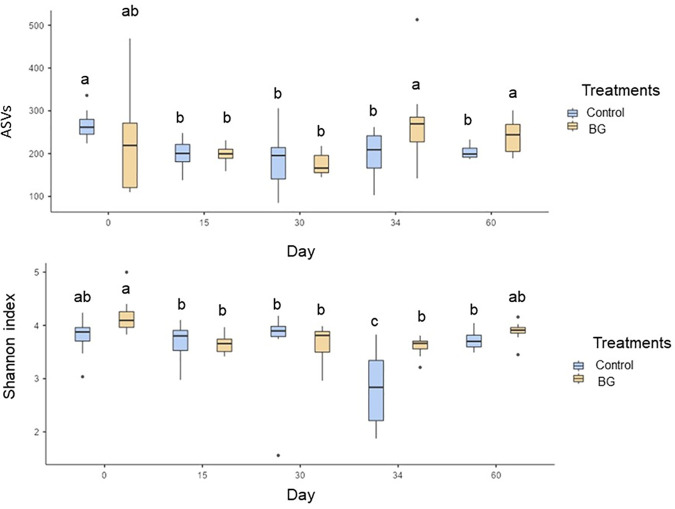
Medians of alpha-diversity indexes (Number of ASVs and Shannon index) of the fecal microbiota of dogs fed without (control) or with BG supplementation on days 0, 15, 30 (before surgery), 34 (after surgery), and 60. Fig 3. ^a,b^Medians followed by distinct letters differ by Dunn’s test (P < 0.05).

The microbiota data showed that the BG group presented an increase in the number of ASVs on days 34 and 60 and in the Shannon index on day 34, compared to the control group (P < 0.05, Fig 3).

**Fig 4:**
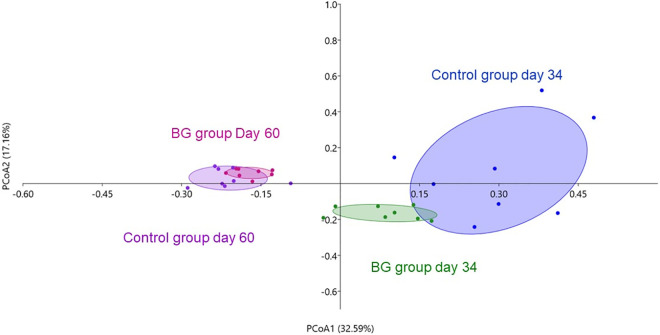
Beta-diversity estimated by Bray-Curtis dissimilarity method indicating the differentiation of fecal bacterial communities of dogs fed without (control) or with BG supplementation on days 34 (after surgery) and 60. Fig 4. P < 0.05 between treatments on each day by PERMANOVA. The figure represents the degree of difference among samples. Each dot represents an animal. PCoA = Principal coordinate analysis.

Beta-diversity analysis indicated a clear differentiation in the general profile of the bacterial communities between the control and BG groups on days 34 (after surgery) and 60 (P < 0.05, Fig 4). Besides, on day 34 (after surgery), the bacterial community profile of the control group was less clustered than the BG group (Fig 4), indicating a more heterogeneous microbiota among dogs. There was no difference in the overall microbiota profile between the groups on the other days (P > 0.05, not shown).

**Fig 5:**
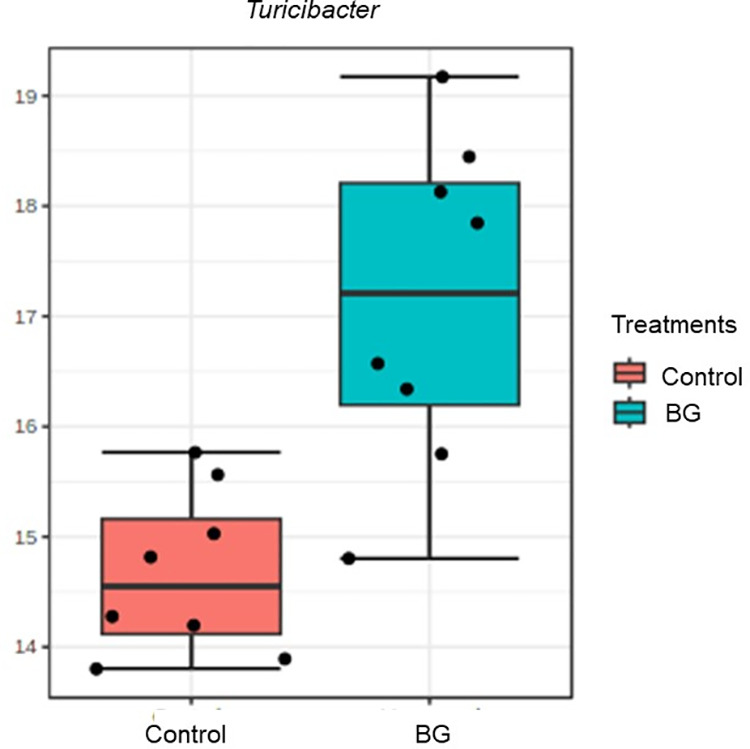
Count (log DNA) of *Turicibacter* in the feces of dogs fed without (control) or with BG supplementation on day 15. Fig 5. According to the LEfSe analysis, *Turicibacter* was significantly enriched in the BG group (LDA score = 4.97). Adjusted P < 0.05 by LEfSe test.

The main phyla and bacterial genera present in the feces of dogs are described in S1 Table and S2 Table, respectively. The LEfSe results indicated that the genus with the greatest discriminatory power between treatments on day 15 was *Turicibacter,* which increased in the BG group (LDA = 4.97, P < 0.05, Fig 5). On day 30, *Megamonas* (LDA = 5.8) and *Gemmiger* (LDA = 3.9) were greater in BG than in the control group (P < 0.05, not shown).

**Fig 6:**
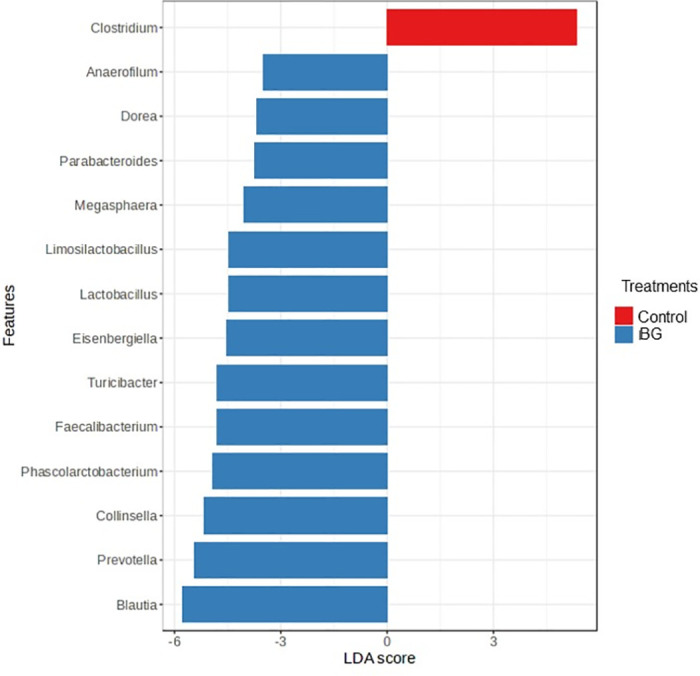
Fecal bacterial genera enriched in the feces of dogs fed without (control) or with BG supplementation on day 34. Fig 6. Linear discriminant analysis (LDA) scores indicate the effect size of each genus. Red bars indicate enrichment in the control group and blue bars indicate enrichment in the BG group. Adjusted P < 0.05 by LEfSe test.

Day 34 (after surgery) was the evaluation period with the greatest differences in the microbiota profile between groups (Fig 6). Among the differences, the genera with the greatest discriminatory power were *Clostridium*, increased in the control group, and the genera *Dorea, Parabacteroides, Megasphaera*, *Limosilactobacillus*, *Lactobacillus*, *Eisenbergiella*, *Turicibacter*, *Faecalibacterium*, *Phascolarctobacterium*, *Collinsella*, *Prevotella*, and *Blautia* increased in the BG group (P < 0.05, Fig 6).

**Fig 7:**
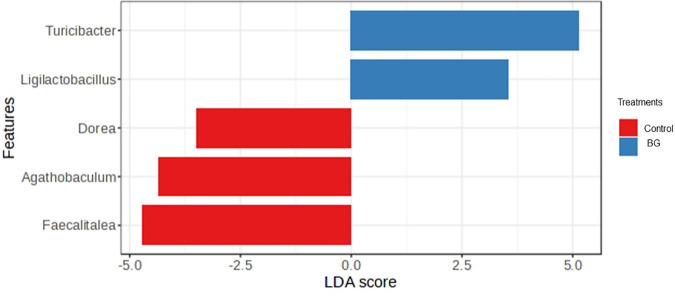
Fecal bacterial genera enriched in the feces of dogs fed without (control) or with BG supplementation on day 60. Fig 7. Linear discriminant analysis (LDA) scores indicate the effect size of each genus. Red bars indicate enrichment in the control group and blue bars indicate enrichment in the BG group. Adjusted P < 0.05 by LEfSe test.

On day 60, the genera with the greatest discriminatory power between groups were *Turicibacter* and *Ligilactobacillus*, increased in the BG group and *Dorea*, *Agathobaculum,* and *Fecalitalea*, increased in the control group (P < 0.05, Fig 7).

### NF-κB and antioxidant enzymes

Serum concentrations of NF-κB increased on day 30 compared to day 0 regardless of treatments (P < 0.05, Table 7).

**Table 7:** Means of antioxidant markers, NF-κB, and intestinal permeability of dogs fed without (control) or with BG supplementation on days 0, 30 (before surgery), 34 (after surgery), and 60.

Treatments^*^	Day	GSH	LPO	SOD	CAT	NF-κB	Perm
**Main effects**							
**Control**		45.9	113	910	8.09	223	0.543
**BG**		62.3	131	873	10.3	227	0.507
	0	47.7	134a	798c	5.08b	190b	0.511ab
	30	55.3	131a	909b	12.0a	265a	0.577a
	34	57.1	142a	1068a	7.16ab	210ab	0.510ab
	60	56.4	80.3b	791c	12.7a	235ab	0.501b
**Interaction**							
**Control**	0	52.2ab	121	824	7.9ab	176	0.533
	30	60.7ab	105	901	8.05ab	258	0.599
	34	53.9ab	137	1102	6.53b	204	0.52
	60	16.90b	87.5	814	9.9ab	254	0.518
**BG**	0	43.1b	147	771	2.25b	205	0.489
	30	49.8ab	157	917	16.0a	271	0.555
	34	60.3ab	146	1034	7.65ab	215	0.5
	60	95.9a	73	769	14.9a	215	0.483
^¹^ **SEM**		4.8	5.82	17.3	0.94	9.5	0.012
**P** ^2^ **-Treatment**		0.008	0.728	0.994	0.019	0.827	0.373
**P** ^2^ **-Day**		0.834	< 0.001	< 0.001	0.002	0.028	0.025
**P** ^2^ **-Treatment x Day**		0.001	0.099	0.416	0.028	0.569	0.965

¹ SEM = standard error of mean; ^2^P = Probability; ^abc^Means with different letters differ according to Tukey’s test (P < 0.05). SOD (U/mL) = superoxide dismutase; CAT (U/mL) = catalase; GSH (pmol/mL) = reduced glutathione; LPO (mmol/mL) = lipid peroxidation; NF-κB (pg/mL); Perm (µg/mL FITC-dextran) = Intestinal permeability; ^*^n = 8/treatment.

The GSH activity was higher in the BG group regardless of the day (P < 0.05, Table 7). There was also an increase in GSH activity in the BG group compared to the control group on day 60 (P < 0.001, Table 7). There was a reduction in serum concentrations of LPO in all dogs on day 60, regardless of treatments (P < 0.05; Table 7).

The SOD enzyme activity was higher on day 34 (after surgery) than on other days, regardless of treatment (P < 0.001; Table 7). The CAT enzyme activity was higher in the BG group than in the control group, regardless of the day (P < 0.05, Table 7). Its activity also increased on days 30 and 60, especially in the group supplemented with BG (P < 0.05, Table 7).

### Intestinal permeability

Both groups presented a reduction in serum concentrations of FITC-dextran on day 60 compared to day 30 (P < 0.05, Table 7).

### Serum-neutralizing antibody titers

The treatments did not influence antibody rabies virus titration, being control = 3.34 UI/ml and BG group = 3.67 UI/ml (P = 0.361, data not shown).

## Discussion

In this study, we used purified BG from *Saccharomyces cerevisiae*. The yeast cell wall is mainly composed of two layers: the outer layer consists of mannan-oligosaccharides (MOS), and the inner layer contains BG. The purification process removes mannoproteins, chitin, and other components of yeast cell walls [35] and exposes the beta-1,3/1,6-glucan structure that interacts with gut cells and confers to BG functional properties distinct from those of other yeast derivatives [36].

Due to differences in molecular structure, such as solubility and chain length, BG may beneficially modulate the gut microbiome and its metabolites, and influence gut functionality via two main mechanisms: (A) immune system recognition, in which BG are detected by specific immune cell receptors, resulting in a direct immune response, which will influence microbiome modulation; and (B) prebiotic mechanism, which BG will be used as a substrate by selective microorganisms in the gut [37,38]. These mechanisms may also be partially attributed to MOS present in yeast cell wall. However, considering that we used purified BG (around 78% BG in the product) and the results of other studies using this specific additive [10,11,39–41], we hypothesize that the effects observed in this study are mainly due to immune system recognition of dogs.

Studies observing the effects of yeast by-products on diet digestibility observed conflicting results in dogs [36]. However, the literature indicates a more consistent pattern when analyzing purified BG, indicating that the low dietary concentration of this additive does not affect nutrient digestibility [10,39], as observed in the current study. The BG supplementation presented no effect on fecal production and consistency, which agrees with a previous study using the same additive in adult dogs [39]. These results are especially relevant for puppies, which frequently undergo stressful situations and require optimized nutrient absorption to ensure adequate growth and mature gastrointestinal systems. Therefore, a supplementation that does not interfere with these parameters is important.

One of the most studied effects of BG is their ability to stimulate the innate immune system, through direct recognition by receptors such as Dectin-1, present on the immune cells’ membrane [42]. These cells recognize BG as molecular patterns associated with microorganisms, triggering a fast and non-specific response that includes phagocytosis, inflammatory response, and “oxidative burst” by activating transcription factors, such as NF-κB [43]. By this mechanism, cells such as monocytes and macrophages undergo epigenetic and metabolic changes in response to inflammation, leading to the production of “trained” innate immune cells, capable of recognizing known or unknown antigens [44].

One of the common methods for assessing the BG action as an enhancer of trained innate immunity is through vaccine response. In the present study, although all the dogs presented seroconversion above 0.5 IU/ml (minimal threshold required), no significant effect of BG supplementation as a vaccine adjuvant was observed. In contrast, other studies have reported that the administration of oral or injectable BG contributes to higher levels of antibodies in dogs after vaccination [40,45]. Although these studies have methodological similarities with the present study, such as the use of the rabies vaccine, a single-dose protocol, and the use of dogs of similar ages (3–4 months), it is important to note that the BG used in those studies was extracted from a different source (*Pleurotus ostreatus*) and administered in different doses, which may lead to distinct biological effects.

A similar study in dogs also reported no effect of BG supplementation on antibody titration [46]. Nevertheless, the group receiving BG in that study reached the protective antibody threshold earlier than the placebo group. The authors suggested that the high immunogenicity of the rabies vaccine and the timing of antibody evaluation may limit the detection of potential adjuvant effects of BG [46]. Considering that, assessing antibody titration at multiple time points would have enabled in the present study a clearer characterization of immune response kinetics, including the timing of threshold achievement and in which group this occurred first.

Along with this innate immune system stimulation, specialized B cells drive adaptive immune responses [44] and produce IgA, the main antibody secreted in the intestinal mucosa, essential for protecting enterocytes against invading microorganisms [47]. In the present study, we observed an increase in fecal IgA after 15 days of BG intake. This effect has already been demonstrated in other species, such as gilts [9]. In dogs, some studies using *Saccharomyces cerevisiae* cell wall components have also observed greater fecal concentrations of IgA [48,49]. However, most studies evaluating purified BG have not reported this effect [10,50,51]. Interestingly, in our study, the increase in fecal IgA was temporary, since on days 30, 34, and 60 we did not observe this result. Two possible hypotheses may explain this result: A) after 15 days of exposure to BG, the immune system responded more robustly initially and remained stable in subsequent days, B) the continuous stimulation of Dectin-1 and TLR2 receptors by BG directs the immune response to T-helper 1 cells, instead of T-helper 2 cells, which stimulate IgA production [52,53]. From a practical perspective, this transient effect may offer benefits in scenarios that demand enhanced mucosal protection, such as during acute infection or periods of heightened vulnerability. Accordingly, further studies are warranted to explore varying BG intake durations and intermittent supplementation strategies aimed at optimizing and sustaining the immune response in dogs.

According to the immune system recognition hypothesis, stimulation of the immune system by BG affects the composition of the intestinal microbiota by modulating the IgA production and the inflammatory response, increasing IL-2 and IL-10, which promotes the development of regulatory T cells [5,42]. In addition, studies in mice indicate that BG can suppress cell signaling that increases the expression of tight junction proteins [54]. Therefore, the inflammatory response is controlled, and the integrity of the epithelial barrier is maintained, preventing the translocation of antigens and bacteria with pathogenic potential [55], and favoring colonization by bacteria related to eubiosis. In this study, although we observed no effect of BG on intestinal permeability, we confirmed that BG supplementation modulated the fecal microbiota and its metabolites. This was evidenced by an increase in SCFA and a reduction in nitrogenous compounds potentially harmful to the intestinal environment. Although these results may suggest a prebiotic effect, it is important to note that studies have identified that purified BG does not affect the production of fermentation metabolites [10,39,56]. Therefore, we suggest that the modulation of these metabolites is probably secondary to the gut microbiota modulation.

On day 15 of BG consumption, we observed a higher abundance of *Turicibacter* and higher fecal concentrations of serotonin. These results may be correlated because *Turicibacter* is involved in the metabolism of serotonin in the intestine, contributing to the regulation of intestinal motility [57]. Also on day 15, we observed an increase in fecal concentrations of spermidine in the BG group that persisted until day 34. Although some amines are related to diseases and damage to mucous membranes [58,59], the threshold between their functional and toxic concentrations for dogs is unknown.

Considering that the general changes in fecal microbiota of the BG group are positive, we suggest that this increase in spermidine may be beneficial. Biogenic amines, such as spermidine, also play important roles in cell proliferation, mucosal turnover, and intestinal epithelial barrier integrity [60–62]. Although no studies were identified specifically evaluating the effect of purified BG on fecal spermidine concentrations, studies with yeast cell wall have also reported an increase in this amine [63]. In contrast, on day 60, we observed a reduction in fecal concentrations of putrescine, histamine, and cadaverine in the BG group, which are amines associated with dysbiosis in humans and dogs [58,59,64]. Although other studies with purified BG have not evaluated these amines, studies with yeast products have also demonstrated a reduction in these compounds in dogs [65].

On day 30 of BG consumption, we observed an increase in bacterial genera associated with the production of SCFA, such as *Megamonas* and *Gemmiger* [66,67]. These results corroborate the higher fecal concentrations of butyrate and the tendency to higher acetate and total SCFA observed in the BG group. SCFA are essential compounds for improving intestinal functionality and their increase is associated with eubiosis in dogs [68,69]. Butyrate is known for its anti-inflammatory effects, by downregulating specific transmembrane receptors and suppressing the production of inflammatory cytokines [70,71].

In this study, all animals underwent surgery and subsequent postoperative antibiotic regimen (amoxicillin with clavulanate) as a standardized physiological challenge to evaluate the modulatory effects of BG supplementation. The impact of surgical stress [2] and use of antibiotics such as amoxicillin with clavulanate on the canine gut microbiota has been well documented, demonstrating reductions in bacterial richness and diversity, along with shifts in overall microbial composition [72]. Consequently, day 34 (three days post-surgery) exhibited the most pronounced alterations in fecal microbiota and its metabolites in both groups, including increased levels of putrescine, phenol, and SOD regardless of treatment. However, these changes were minimized by the BG supplementation [73].

In dogs, one of the characteristics of eubiosis is the high diversity of the intestinal microbiota [74]. We observed that the consumption of BG promoted an increase in alpha diversity indexes. This result is particularly interesting on day 34, demonstrating that BG was able to maintain the diversity of the fecal microbiota, even after surgical challenge and the use of antibiotics. This effect of BG supplementation was also observed in pre-weaned pigs [75]. In dogs, [10] reported no effect of BG supplementation on fecal microbiota alpha diversity. Despite this, a study using BG derived from the *H. erinaceus* mushroom demonstrated a tendency for greater alpha diversity of the fecal microbiota in dogs supplemented with this additive. These results demonstrate the BG’s modulatory potential on gut microbiota of dogs, even if derived from different sources [76].

Assessing the overall profile of fecal bacterial communities, the beta-diversity analysis indicated different microbiota compositions due to BG supplementation, both on day 34 (after castration) and on day 60. Interestingly, on day 34, the fecal microbiota of dogs of the control group was less clustered, which means that the microbiota was less similar among individuals of the same group, compared to the BG group. This indicates a possible contribution of BG to the stabilization of gut microbiota after the surgical challenge. This hypothesis was confirmed by LEfSe analysis, which identified the presence of genera such *as Blautia, Faecalibacterium,* and *Turicibacter* in the BG group. In addition to presenting important metabolic functions, these genera are considered sentinels of the intestinal microbiota, and their increase is considered a biomarker of eubiosis in dogs [77]. Specifically, *Faecalibacterium* is known for its anti-inflammatory properties in the colonic mucosa, mostly through the production of butyrate [78]. Also, the *Blautia* genus is a propionate producer [68]. Decreased faecal concentrations of these genera has been reported in dogs with acute haemorrhagic diarrhoea, chronic enteropathy, and inflammatory bowel disease [79, 80], supporting the association between higher abundance of these taxa and improved intestinal health in dogs. In contrast, the genus with the greatest discriminatory power in the control group after surgery was *Clostridium*. Although we did not evaluate species level, we can suggest that the *Clostridium* species may be of pathogenic potential, since the intestinal environment of the control group presented higher concentrations of nitrogen compounds on day 34.

Other bacteria also predominated in the BG group on day 34 were *Lactobacillus* and *Lysimosilactobacillus*, which are lactic acid producers and are commonly used as probiotics for dogs [81–83]. In addition, we observed the presence of the genus *Prevotella* in the BG group, a SCFA producer associated with eubiosis in dogs [69,80,84]. One of the few studies that evaluated the effects of purified BG on the fecal microbiota of dogs also observed an increase in *Faecalibacterium* and *Prevotella* in the supplemented group [10]. The increase in these genera may have contributed to the tendency to lower fecal pH observed in the BG group. This acidified environment is a natural barrier against the growth of potentially pathogenic microorganisms in the gut [85].

Although we observed positive effects related to a possible modulation of the inflammatory response in the BG group, we did not observe any effect on NF-κB. This may be related to the use of anti-inflammatory drugs in the post-surgical period, which are known to inhibit NF-κB activity [86], interfering with the secretion of acute phase proteins. Nonetheless, its potential anti-inflammatory activity may still be revealed through alternative biomarkers not examined in the present study (e.g., cytokines), which would be explored in future research.

BG have “scavenging activity” due to the presence of hydroxyl groups capable of binding to metal ions, inhibiting the formation of free radicals [87]. Although we did not observe an effect on LPO, there was an increase in catalase and GSH activities, suggesting that BG may stimulate the endogenous antioxidant system. Despite the exact mechanism of this stimulation is not yet completely elucidated, similar results have been described in studies with *S. cerevisiae* yeast cell wall in pigs and poultry [88,89]. These findings are clinically relevant, as increased oxidative stress and reduced endogenous antioxidants—such as GSH—have been reported in dogs with systemic diseases (e.g., anemia, congestive heart failure) and in hospitalized patients [90–92]. In contrast, elevated GSH levels observed in the BG group may indicate enhanced antioxidant defense and cellular protection, which is particularly beneficial during physiologically stressful periods, such as growth, environmental transitions, or surgical interventions.

Finally, as far as we know, this is the first study to evaluate and obtain significant results from BG on oxidative markers and intestinal microbiota of puppies subjected to surgical challenge, providing important insights into the benefits of these purified molecules.

This study presents some limitations, such as the relatively low number of replicates per treatment. Moreover, it is important to emphasize that, although the mechanisms proposed in this discussion remain biologically plausible, they should be interpreted with caution. Further studies are necessary to confirm these findings using additional markers—such as serum cytokine concentrations, receptor expression, or functional cellular responses (e.g., leukocyte and monocyte counts, phagocytic index)—as well as clinical outcomes that may more accurately reflect the specific effects of BG on the canine innate immune system.

## Conclusions

BG purified from the yeast *Saccharomyces cerevisiae* can be used as a dietary supplement for puppy dogs at 65 g/kg body weight, modulating the immune system, as evidenced by an increase in fecal IgA after 15 days of supplementation. Additionally, it also positively influenced the intestinal microbiome of puppies by promoting the growth of bacteria associated with eubiosis, such as *Turicibacter*, *Faecalibacterium*, and *Blautia*, and improving the intestinal environment through increased butyrate, serotonin, and spermidine fecal concentrations. Additionally, BG supplementation led to less variation in fecal bacterial communities and sustained higher microbial richness after the surgical challenge and demonstrated antioxidant effects by increasing GSH and catalase activities in puppies.

## Supporting information

S1 Table Medians and interquartile range (IQR) of most abundant phyla (%) in the feces of dogs fed without (control) or with supplementation of BG on days 0, 15, 30 (before surgery), 34 (after surgery) and 60. S1 Table. C = control group.
(TIFF)

S2 Table Medians and interquartile range (IQR) of most abundant genera (%) in the feces of dogs fed without (control) or with supplementation of BG on days 0, 15, 30 (before surgery), 34 (after surgery) and 60. S2 Table. C = control group.
(TIFF)
