# Supplementary figures and images for: Effects of yeast beta-1,3/1,6-glucans on nutrient digestibility, intestinal functionality, and immune and antioxidant variables in growing dogs submitted to spay or neutering surgery
Source: PLoS One. 2025 Sep 8;20(9):e0331843. doi: 10.1371/journal.pone.0331843 (PMC12416722; doi:10.1371/journal.pone.0331843)

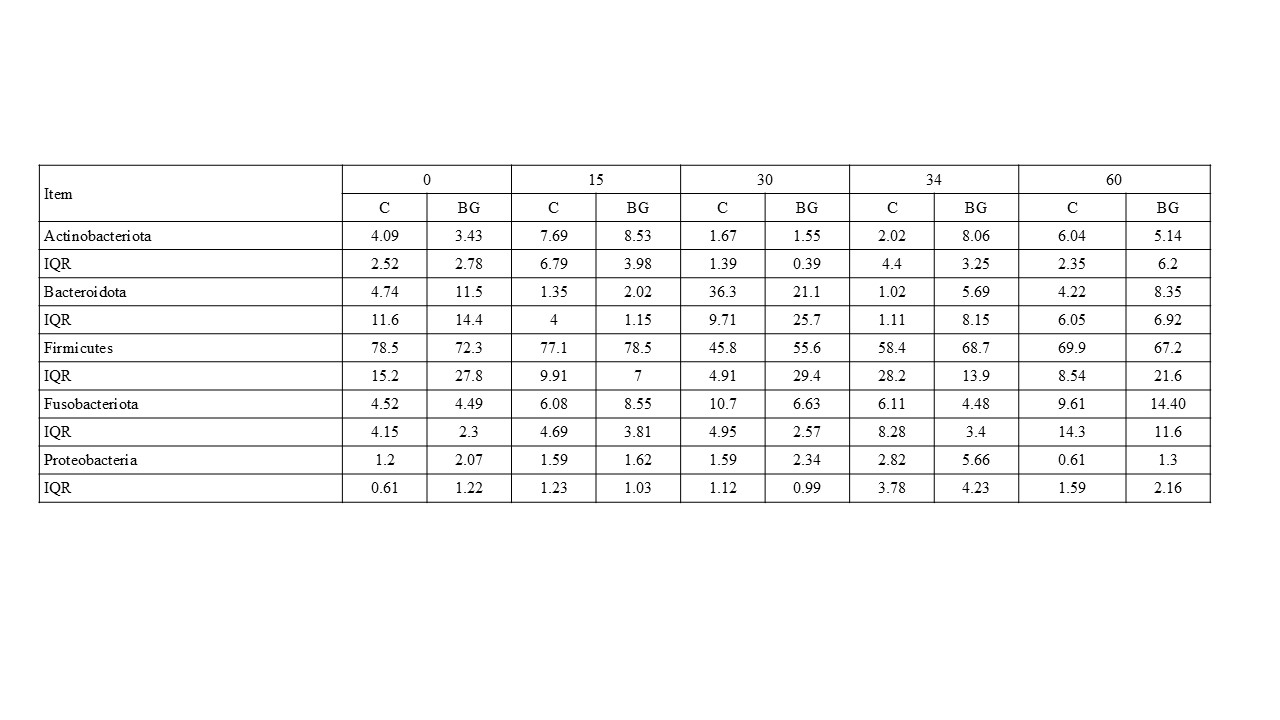

Supplement: S1 Table — C = control group. (TIFF) [file pone.0331843.s001.tiff]

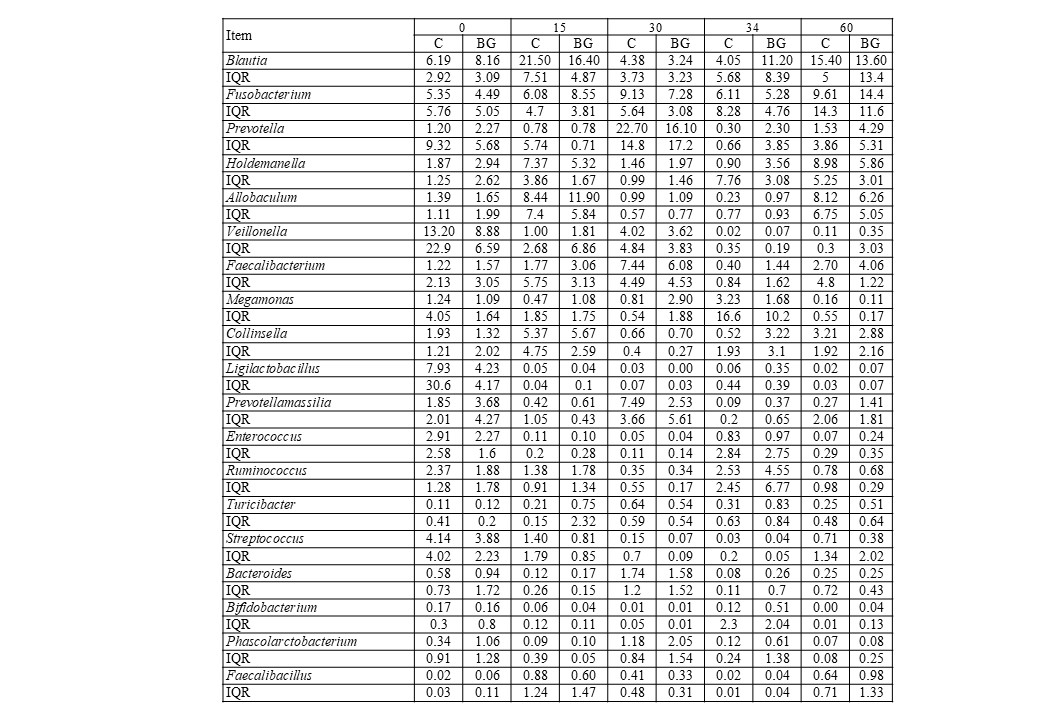

Supplement: S2 Table — C = control group. (TIFF) [file pone.0331843.s002.tiff]
